# Serum CXCL5 Detects Early Hepatocellular Carcinoma and Indicates Tumor Progression

**DOI:** 10.3390/ijms24065295

**Published:** 2023-03-10

**Authors:** Alena Laschtowitz, Joeri Lambrecht, Tobias Puengel, Frank Tacke, Raphael Mohr

**Affiliations:** 1 Department of Hepatology & Gastroenterology, Campus Virchow-Klinikum and Campus Charité Mitte, Charité-Universitätsmedizin Berlin, 13353 Berlin, Germany; 2 Berlin Institute of Health, 10178 Berlin, Germany

**Keywords:** hepatocellular carcinoma (HCC), chemokines, biomarker, cirrhosis, etiology, C-X-C motif chemokine ligand (CXCL) 5, CXCL9, CXCL10

## Abstract

Chemokines or chemotactic cytokines play a pivotal role in the immune pathogenesis of liver cirrhosis and hepatocellular carcinoma (HCC). Nevertheless, comprehensive cytokine profiling data across different etiologies of liver diseases are lacking. Chemokines might serve as diagnostic and prognostic biomarkers. In our study, we analyzed serum concentrations of 12 inflammation-related chemokines in a cohort of patients (*n* = 222) with cirrhosis of different etiologies and/or HCC. We compared 97 patients with cirrhosis and treatment-naïve HCC to the chemokine profile of 125 patients with cirrhosis but confirmed absence of HCC. Nine out of twelve chemokines were significantly elevated in sera of cirrhotic patients with HCC compared to HCC-free cirrhosis controls (CCL2, CCL11, CCL17, CCL20, CXCL1, CXCL5, CXCL9, CXCL10, CXCL11). Among those, CXCL5, CXCL9, CXCL10, and CXCL11 were significantly elevated in patients with early HCC according to the Barcelona Clinic Liver Cancer (BCLC) stages 0/A compared to cirrhotic controls without HCC. In patients with HCC, CXCL5 serum levels were associated with tumor progression, and levels of CCL20 and CXCL8 with macrovascular invasion. Importantly, our study identified CXCL5, CXCL9, and CXCL10 as universal HCC markers, independent from underlying etiology of cirrhosis. In conclusion, regardless of the underlying liver disease, patients with cirrhosis share an HCC-specific chemokine profile. CXCL5 may serve as a diagnostic biomarker in cirrhotic patients for early HCC detection as well as for tumor progression.

## 1. Introduction

Hepatocellular carcinoma (HCC) is a heterogeneous disease that usually arises in chronically inflamed or cirrhotic livers induced by multiple-etiology-specific pathomechanisms [1]. Besides chronic viral infections (predominantly hepatitis B (HBV) and C (HCV) virus), alcohol-related liver disease (ALD), and non-alcoholic fatty liver disease (NAFLD), autoimmune, cholestatic, and hereditary liver diseases are drivers for chronic liver injury [2]. These conditions present a chronic stimulation of the immune system, contributing to persistent hepatic inflammation and hereby providing the seeding ground for hepatocarcinogenesis [3]. The complex immunological responses driving the progression from chronic liver injury to cirrhosis and cancer are influenced by a variety of factors including cytokines and the local microenvironment [4].

Cytokines display a heterogenous group of functional proteins, including chemokines and interleukins, maintaining pivotal roles in the regulation of cell signaling. Cell death mechanisms (e.g., necrosis, ferroptosis) and activated immune cells (predominantly phagocytes) represent the major source of cytokines as a response to liver injury and inflammatory stimuli [5,6]. Tumor-promoting inflammatory processes additionally comprise cytokines released from premalignant and tumor cells leading to the recruitment of immune cells, which in turn specifically shape and further aggravate the tumor microenvironment (TME). This promotes oncogenic transformation and tumor growth, as well as facilitating invasion and metastasis [7,8]. Although a large number of different cytokines have been identified in the context of chronic liver injury, the role of individual cytokines and their interrelation with highly plastic immune cells in the TME as well as their distinct relevance in the development and progression of cirrhosis or HCC remains less clear [9]. Nevertheless, recent mechanistic studies are providing further insight into the mechanisms of action of individual chemokines. Accordingly, it could be shown that CXCL10 modulates the infiltration of anti-tumorigenic immune cells in HCC while CXCL5 promotes proliferation, migration, and invasion of HCC cells through the activation of PI3K and ERK1/2 signaling pathways with consecutive infiltration of neutrophils [9,10]. In the setting of HCC, previous studies mainly focused on viral induced liver disease and identified serum cytokine profiles that were associated with the risk of carcinogenesis [11,12,13]. However, it remained unclear if those HCC-discriminant biomarkers also add value in other viral etiologies that are viral, as well as if they correlate with HCC progression. Most recently, a pilot study found etiology-specific cytokine panels for the detection of early HCC [14]. Interestingly, no common HCC-discriminant chemokines shared between all etiologies could be identified, although former data indicate common hepatocarcinogenetic chemokine pathways [15].

On the basis of these findings, we hypothesized that (i) chemokines may not only be associated with presence of early HCC but also mirror tumor progression in HCC patients and that (ii) in addition to etiology-specific cytokine panels, a generalized set of chemokines might be able to detect HCC development in patients with cirrhosis, regardless of the underlying liver disease. In this study, we investigated serum levels of 12 pro-inflammatory chemokines in patients with cirrhosis of different underlying etiologies (ALD, NAFLD, viral hepatitis, autoimmune/cholestatic liver diseases) with or without HCC.

## 2. Results

### 2.1. Baseline Patient Characteristics

Tumor-promoting inflammatory processes including complex intercellular and cytokine signaling are a hallmark of HCC [16]. Therefore, characterization of distinct cytokine profiles potentially serves as diagnostic or prognostic tools for HCC detection [12]. To identify a preferably HCC-specific chemokine panel and exclude potential confounding factors, we specifically included 97 cirrhotic patients with treatment-naïve HCC, referred to as HCC patients in the present manuscript. As a control group, we analyzed blood samples from 125 cirrhotic HCC-free patients. Biochemical and clinical features of all patients are detailed in Table 1. Distribution of sex was similar between the groups, while the patients with HCC were significantly older than the control group (median years = 66 (43–85) vs. 54 (25–71), *p <* 0.001). In the HCC group, a greater proportion showed a compensated cirrhosis (Child–Pugh A) compared to the control group (Child–Pugh A: 77.3% vs. 42.3%, *p <* 0.001), while none presented with advanced Child–Pugh C stage.

### 2.2. Identification of an HCC-Discriminant Chemokine Panel

**Figure 1:**
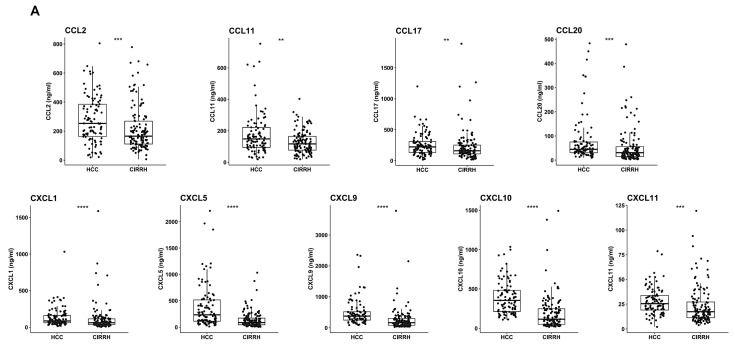

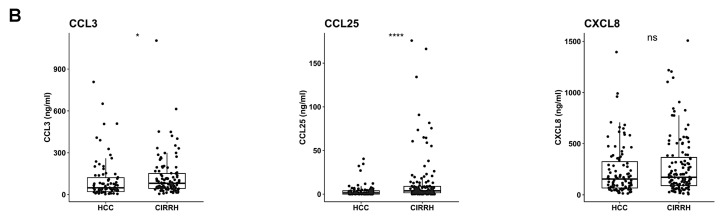
Inflammatory chemokine serum levels in patients with HCC or liver cirrhosis. Scatter dot plots with horizontal bars indicating median and IQR (ns *p >* 0.05, * *p ≤* 0.05, ** *p ≤* 0.01, *** *p ≤* 0.001, **** *p ≤* 0.0001). (**A**) Chemokines significantly upregulated in patients with HCC compared to cirrhotic patients without HCC. (**B**) Chemokines significantly downregulated in patients with HCC or without difference compared to cirrhotic patients without HCC. Abbreviations: CCL, chemokine C-C motif ligand; CIRRH, cirrhosis; CXCL, C-X-C motif chemokine ligand; HCC, hepatocellular carcinoma.

The composition of various cytokines during the course of chronic liver disease has been extensively investigated in the context of different etiologies [12]. Yet, cytokine signaling during HCC initiation, promotion, and progression is less well described [7,12]. In a first step, we investigated the association of serum levels of 12 pro-inflammatory chemokines with the presence of HCC: In HCC patients, 9 out of 12 chemokines showed significantly increased serum levels compared to cirrhotic patients without HCC (CCL2, CCL11, CCL17, CCL20, CXCL1, CXCL5, CXCL9, CXCL10, CXCL11) (Figure 1A). Only the chemokines CCL3 and CCL25 were significantly decreased in sera of HCC patients compared to cirrhotic patients. Interestingly, serum levels of CXCL8 (IL-8) showed no significant difference between the cirrhotic patients with or without HCC (Figure 1B).

To avoid the possibility that decompensated cirrhosis may obscure the differences of circulating chemokines between cirrhotic patients with or without HCC [17,18,19], we excluded patients with decompensated cirrhosis (Child–Pugh Score C) from our analysis. Exclusion of patients with decompensated cirrhosis did not alter statistical differences in chemokine serum levels between the cirrhotic patients with or without HCC. In the group of patients with compensated cirrhosis and HCC (*n* = 96), the same nine chemokines still showed significantly increased serum levels when compared to patients with compensated cirrhosis without HCC (*n* = 110). While CXCL8 (IL-8) concentrations showed no difference between the two groups, CCL3 and CCL25 serum levels were significantly higher in HCC-free cirrhotic patients.

Previous reports pointed at specific circulating cytokine profiles that may be indicative for the presence of HCC at a very early stage of its development, suggesting a potential role for cytokines as diagnostic biomarkers [11,12,13,14]. Accordingly, we evaluated whether the formerly identified HCC-discriminant chemokines were robust in early HCC stages, defined as BCLC 0 (very early stage) and BCLC A (early stage), when compared to sera of HCC-free cirrhotic controls. Indeed, four out of nine chemokines (CXCL5, CXCL9, CXCL10, CXCL11) were significantly elevated in sera of patients with early HCC compared to cirrhotic patients (median CXCL5 ng/mL = 191 (82–1156) vs. 88 (6–1032), *p <* 0.001; CXCL9 ng/mL = 359 (88–2325) vs. 165 (29–3782), *p <* 0.001; CXCL10 ng/mL = 295 (139–999) vs. 116 (19–1493), *p <* 0.001; CXCL11 ng/mL = 26 (2–79) vs. 17 (4–120), *p =* 0.044).

### 2.3. Chemokines Associated with Tumor Stage and Spread

**Figure 2:**
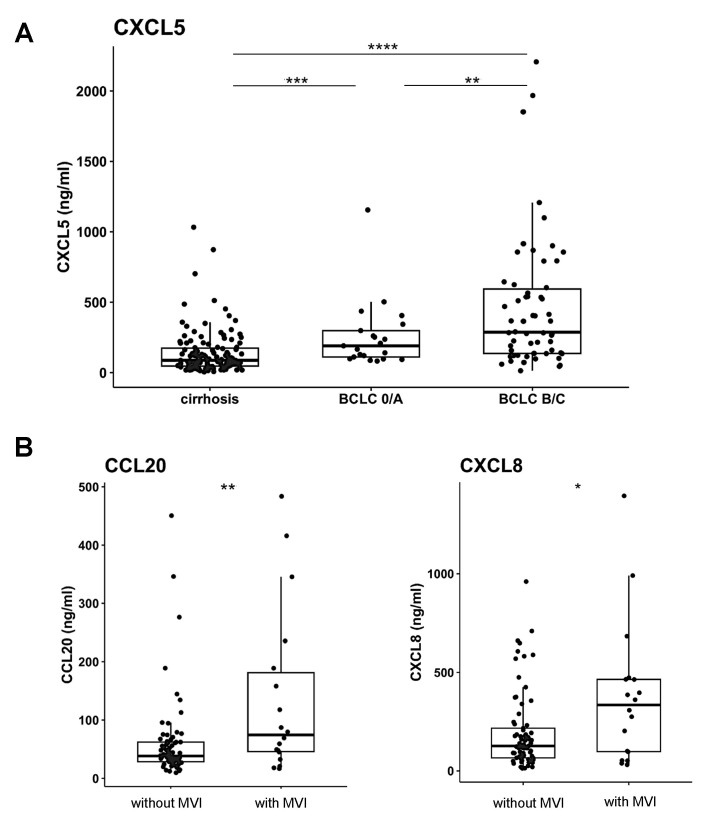
Inflammatory chemokine serum levels in patients with cirrhosis and with early or advanced HCC. Scatter dot plots with horizontal bars indicating median and IQR (* *p ≤* 0.05, ** *p ≤* 0.01, *** *p ≤* 0.001, **** *p ≤* 0.0001). (**A**) CXCL5 was significantly upregulated in patients with advanced HCC compared to early HCC and patients with cirrhosis but without HCC. (**B**) CCL20 and CXCL8 were significantly elevated in HCC patients with MVI compared to HCC patients without MVI. Abbreviations: CCL, chemokine C-C motif ligand; CXCL, C-X-C motif chemokine ligand; MVI, macrovascular invasion.

Given that chemokines might serve as marker of early HCC detection, we aimed to evaluate whether these chemokines might also be associated with tumor progression. To assess this possible association, we compared patients with early HCC (BCLC 0/A) and more advanced HCC (BCLC B/C). Only CXCL5 serum levels remained significantly increased in patients with more advanced vs. early HCC (CXCL5 ng/mL = 287 (14–2206) vs. 191 (82–1156), *p* = 0.049) (Figure 2A). Moreover, CCL20 and CXCL8 were associated with macrovascular invasion (MVI), being significantly increased in HCC patients with MVI compared to HCC patients without MVI (CCL20 ng/mL = 74 (17–484) vs. 38 (10–450), *p =* 0.013; CXCL8 ng/mL = 335 (31–1395) vs. 127 (13–960), *p* = 0.04) (Figure 2B).

### 2.4. Etiology-Specific and Common Chemokine Profiles in HCC

As previous reports emphasized the impact of underlying etiology on the circulating cytokine profiles [12,14], we compared the chemokine composition in patients with or without HCC according to etiology (ALD, NAFLD, viral, cholestatic/autoimmune, others). Indeed, there was a common HCC-associated chemokine profile across underlying etiologies. For each etiology-based subgroup (NAFLD, ALD, viral hepatitis, and “other”), CXCL5, CXCL9, and CXCL10 were significantly elevated in HCC patients and might be used as etiology-independent HCC-discriminant chemokine panel (Table 2). In the subgroup of cholestatic/autoimmune liver disease, CXCL9 lacked significance between the two subgroups. This might have been due to the small sample size of this group.

## 3. Discussion

Despite recent progress in the clinical management of chronic liver diseases, prognosis of HCC remains poor due to the fact that HCC is often diagnosed when curative therapies (i.e., resection, liver transplantation, local ablative techniques) are not feasible anymore or liver function is already too compromised [20]. Prognosis is jointly determined by the degree of liver cirrhosis and the tumor stage. Solely in early stage disease, curative treatment options may be offered. Surgical resection is the curative treatment of choice. Patients with limited tumor burden may also be considered for liver transplantation. However, the majority of patients are diagnosed with intermediate or advanced tumor stages, wherein merely palliative treatment options are feasible. There is an unmet need for novel HCC screening options to enable early diagnosis and further improve patients’ prognosis.

Recently, the role of inflammatory chemokines has been demonstrated in the context of chronic liver diseases including HCC of different etiologies [11,13,21]. They are known drivers of liver cirrhosis and HCC development as well as progression. In addition to ubiquitous pro-fibrogenic and pro-oncogenic mechanisms, etiology-specific chemokine profiles also appear to play a role [14]. In this context, chemokines represent an important tool to gain new insights into the mechanisms of hepatocarcinogenesis. Furthermore, they have the potential to serve as diagnostic and prognostic biomarkers for improved patient stratification. By analyzing a well-characterized cohort of cirrhotic patients with HCC and a control group of cirrhosis with confirmed absence of HCC, we demonstrated that a biologically plausible set of chemokines consisting of CCL2, CCL11, CCL17, CCL20, CXCL1, CXCL5, CXCL9, CXCL10, and CXCL11 might be a useful tool for the discrimination between cirrhosis and HCC development.

Most interestingly, this difference of soluble chemokine levels between early HCC (defined as BCLC 0/A) compared to cirrhotic control patients remained consistent in four out of nine chemokines (CXCL5, CXCL9, CXCL10, and CXCL11). CXCL10 regulates several hallmarks of the tumor microenvironment and especially modulates the infiltration of anti-tumoral T cells [10]. CXCL11 was described as a marker of liver injury caused by viral hepatitis [22] and was even positively associated with development of HCC compared to non-cirrhotic controls in patients with chronic HCV and HBV infection [13]. In addition to the potential diagnostic value, chemokines might also serve as a progression marker in HCC. Serum levels of CXCL5 were significantly higher in intermediate or advanced disease stages (BCLC B/C) when compared to early stage disease (BCLC 0/A), indicating a role for this chemokine as potential biomarkers for tumor progression and spread in cirrhotic patients. This result is in line with previous findings that suggested a pivotal role of CXCL5 in HCC development and progression [23,24,25,26]. CXCL9 is released by parenchymal (e.g., hepatocytes, liver sinusoidal endothelial cells) as well as non-parenchymal (e.g., macrophages, hepatic stellate cells (HSCs)) immune cells and can be linked together with its main receptor CXCR3 to lymphoid immune responses upon liver injury of different etiologies [22,27,28]. However, the distinct role of CXCL9 remains less clear as experimental studies report direct angiostatic and antifibrotic effects of the CXCL9/CXCR3 under pre-cirrhotic conditions [29], while elevated CXCL9 serum levels potentially contribute to hepatic as well as extrahepatic organ dysfunction in cirrhotic patients predicting poor overall survival [30]. In HCC, CXCL9 is associated with the recruitment of tumor-infiltrating immune cells (mainly CD4+ and CD8+ lymphocytes) shaping the TME and possibly amplifying anti-tumor responses of cytotoxic T cells [31]. Former studies identified different cytokines associated with HCC development in different underlying liver diseases. Nevertheless, no common panel could be identified. A recent study illuminates the impact of etiology on circulating immune mediators [14]. Unlike previous reports, we were able to identify CXCL5, CXCL9, and CXCL10 as universal HCC markers independent from underlying etiology. Only in our subgroup of patients with cholestatic or autoimmune liver diseases did CXCL9 drop out, most likely due to the small sample size. Similar to our data, CXCL9 increase was previously associated with HCC development in the context of NAFLD [14] and HCV infection [11]. To the best of our knowledge, our study provides the first evidence on an HCC-discriminant chemokine profile shared by all etiologies.

Furthermore, we report that high CCL20 and CXCL8 serum levels were associated with microvascular invasion, a characteristic of advanced tumor stages. In chronic liver disease, CCL20 (a ligand of CCR6) promotes inflammation and fibrogenesis via γδ T cells and HSCs responses [21]. In line with our observations, the CCL20/CCR6 axis has been directly linked to promote carcinogenesis in several cancer entities including primary liver cancers by re-shaping the immunologic TME and subsequently migration as well as proliferation of cancer cells [32]. The role of CXCL8 has been described extensively in the development and progression of liver cancers, mediating the recruitment of neutrophils to the tumor site and favoring tumor invasion, for instance, via secretion of matrix metalloproteinases, thus indicating poor prognosis [33]. Therefore, one could speculate that both markers, CCL20 and CXCL8, discriminate advanced tumor stages rather than detecting early HCC and predict poor prognosis.

Monocytes and macrophages are key players orchestrating complex inflammatory immune responses in chronic liver disease and in consequence stimulate HSC activation and transdifferentiation towards the extracellular matrix (ECM), producing myofibroblast—a crucial step in the development of hepatic fibrosis and HCC [34]. In this context, (resident) macrophages represent the major source of cytokines and chemokines upon liver injury, attracting further immune cells and shaping the local microenvironment [35]. Recent studies highlight the heterogeneity as well as the disease-specific, context-dependent plasticity of phagocytes and their functional consequences [36]. Therefore, during carcinogenesis, the pro- and anti-inflammatory properties of macrophages can specifically shape the TME but can also be exploited by the tumor [37]. CCL2 is considered to be the predominant chemokine for the recruitment of monocytes and macrophages to the site of liver injury. In addition, the CCL2/CCR2 axis plays a pivotal role in the macrophage-mediated immunosuppression status of the TME [38,39]. In mouse models, CCL2 and CCL9 were shown to contribute to the mobilization of myeloid-derived suppressor cells, potentially facilitating tumor growth [40]. Tumor-cell-derived CCL20 interacts with B lymphocytes and promotes HCC progression via enhancing angiogenesis [41]. This pro-angiogenic effect was shown to be directly induced by HCV [42]. CCL11 was previously associated with the development of cirrhosis in the context of chronic HCV infections [43]. Aberrant expression of the macrophage inflammatory protein-1 alpha/CCL3 as well as CCL17 were previously described in human HCC tissues [44,45]. CCL25 may contribute to the pathogenesis of NAFLD [46]. Data from both in vivo and in vitro models showed that the CXCR2/CXCL1 axis as well as the CXCR2/CXCL5 axis regulate neutrophil infiltration into tumor tissues, inducing epithelial–mesenchymal transition and thus aggravating malignant behavior of HCC cells [24,47,48].

While we believe the present study may show the potential of evaluating serum cytokines in the clinical context of HCC, we acknowledge several limitations, mainly due to the retrospective and single-center study design. Prospective validation in a larger multi-center study would be required to represent the spectrum of inflammatory cytokines in HCC progression at a larger scale. We provide a well-characterized patient cohort comprising a variety of the underlying disease etiologies, which realistically reflects the real-life situation, but subanalysis of etiology-specific chemokines was restricted to the heterogeneity-based small group sizes, limiting statistical analysis. Since our data and previous studies have shown the importance of etiology-specific mechanisms, future studies should focus on chemokine mechanisms also in rare liver diseases. It should also be taken into account that a selective chemokine panel based on the current literature and knowledge cannot fully reflect the complexity of the human situation in HCC. Moreover, our current study lacks additional analyses that would further strengthen the interpretation of our data set. Future studies might include histological assessment as well as spatial transcriptomic or proteomic analyses.

Immune regulation is pivotal in the microenvironment of HCC. Nevertheless, the specific players and interactions seem only rudimentarily understood. Further mechanistical investigation is urgently needed in order to improve the potential of inflammatory cytokines as biomarkers for early disease diagnosis and to prospectively develop new therapeutic options for patients with HCC. Especially in the era of checkpoint inhibitor therapies, inflammatory cytokines might have the capacity to evaluate early treatment responses potentially guiding treatment decisions.

## 4. Materials and Methods

### 4.1. Patient Population

This retrospective single-center study included a total of 222 patients with cirrhosis of different etiologies treated at the Department of Hepatology and Gastroenterology at Charité–Universitätsmedizin Berlin. Among these, serum samples of 97 patients with cirrhosis and concomitant HCC, and from 125 patients with cirrhosis but confirmed absence of HCC, were included.

The diagnosis of cirrhosis and the underlying chronic liver disease was based on clinical, biochemical, serological, radiological, and histopathological findings according to current guidelines [49,50,51,52,53,54,55,56]. The diagnosis of HCC was confirmed according to the European Society of Medical Oncology (ESMO) criteria by histologic analysis or clinical and imaging features for patients with liver cirrhosis [57]. HCC classification was made according to the current Barcelona Clinic Liver Cancer (BCLC) recommendation [58]. All etiologies of liver cirrhosis and all stages of treatment-naïve HCC were accepted for study inclusion.

### 4.2. Clinical and Biochemical Parameters

We assessed routine biochemical parameters and clinical signs of hepatic decompensation. Clinical data were collected from medical records at presentation. Blood samples from HCC patients were collected at tumor diagnosis, prior to any tumor specific therapy. HCC stage was assessed by computed tomography and/or magnetic resonance imaging. For analysis, patients with drug induced liver injury, hemochromatosis, and cryptogenic liver disease were combined together in one group.

### 4.3. Assay Methods

Serum samples were obtained through centrifugation for 10 min at 2000× *g* and were then stored at −80 °C until use. Evaluation of cytokine content in serum samples was performed using the Human Proinflammatory Chemokine Panel 1 (12-plex) with V-Bottom Plate (Biolegend, San Diego, CA, USA) according to the manufacturer’s protocol using technical duplicates. Measurements were performed using a BD FACSCanto™ II (BD Biosciences, Franklin Lakes, NJ, USA) with standard settings in the APC and PE channels. The Human Proinflammatory Chemokine Panel 1 includes the following chemokines: CCL2 (MCP-1), CCL3 (MIP-1a), CCL5 (RANTES), CCL11 (Eotaxin), CCL17 (TARC), CCL20 (MIP-3a), CCL25, CXCL1 (GROa), CXCL5 (ENA-78), CCL9 (MIG), CXCL8 (IL-8), CXCL10 (IP-10), and CXCL11 (I-TAC). Concentrations were given as Median (Interquartile Range (IQR)) in ng/mL.

### 4.4. Statistical Analysis

Percentages and counts are provided for categorical data. Median values with the corresponding range were calculated for continuous data. To test for differences between groups, non-parametric tests, including the Wilcoxon signed rank test, were performed. A comparison of categorical data between groups was performed using the Pearson’s chi-square test. All *p*-values were two-tailed; *p* < 0.05 was considered statistically significant. Figure design and statistical testing were carried out using R Version 3.6.0 and R Studio Version 1.2.1335.

## Figures and Tables

**Table 1:** Biochemical and clinical features of all patients with cirrhosis with or without HCC.

Feature	Patients with HCC (*n* = 97)	Patients with Cirrhosis (*n* = 125)	* *p*-Value
Sex, female, *n* (%)	21 (22%)	40 (32%)	0.087
Age median (range), years	66 (43–85)	54 (25–71)	<0.001
Child Pugh Score, % (*n*) A B C	77.3% (75) 21.6% (21) - NA = 1	42.4% (53) 45.6% (57) 8% (10) NA = 5	<0.001
MELD score median (range)	9.7 (5.7–25.3)	14.3 (6.4–36.6)	<0.001
ALBI, % (*n*) Grade 1 Grade 2 Grade 3	37.1% (36) 50.5% (49) 11.3% (11)	15.8% (16) 49.5% (50) 34.6% (35)	<0.001
ALT median (range), U/L	44 (6–219)	34 (6–392)	0.012
Albumin median (range), g/L	37.5 (1.0–51.2)	33 (2.8–48)	<0.001
Bilirubin median (range), mg/dL	1.1 (0.1–10)	2 (0.3–29.1)	<0.001
Creatinin median (range), mg/dL	0.8 (0.4–2)	0.9 (0.5–4.3)	0.003
INR (range)	1.2 (0.9–2.1)	1.4 (0.8–4.6)	<0.001
BCLC, % (*n*) 0 A B C NA	10% (8) 16.2% (13) 43.8% (35) 30% (24) 17.5% (17)	-	-
Macrovascular invasion in HCC, % (*n*) NA	18.5% (18) 4% (4)		
Underlying etiology, % (*n*)			0.046
Viral hepatitis	32% (31)	19.2% (24)	
Alcohol	34% (33)	35.2% (44)	
NAFLD	21.6% (21)	24% (30)	
Cholestatic/autoimmune (PBC, PSC, AIH)	4.1% (4)	10.4% (13)	
Other origin	8.2% (8)	10.4% (13)	

Median values are presented with interquartile range in brackets. Percentages are given with total numbers in brackets. Abbreviations: AIH, autoimmune hepatitis; ALBI, albumin–bilirubin; ALT, alanine aminotransferase; BCLC, Barcelona Clinic Liver Cancer; INR, international normalized ratio; MELD, model for end-stage liver disease; NAFLD, non-alcoholic fatty liver disease; PBC, primary biliary cholangitis; PSC, primary sclerosing cholangitis. * Continuous variables were compared using Wilcoxon signed rank test. Pearson’s chi-squared test was used for comparing percentages.

**Table 2:** Chemokines with significant difference in cirrhotic patients with or without HCC due to different etiologies.

	**Viral Cirrhosis with HCC (*n* = 31)**	**Viral Cirrhosis (*n* = 23)**	***p*-Value**
CCL25	0.8 (0–34.6)	3.5 (0–90.8)	0.002
CXCL5	190.3 (13.5–1967.2)	74.7 (5.5–1032.7)	<0.001
CXCL9	393.8 (158–1316.5)	269.1 (42.8–2150.7)	0.025
CXCL10	482.1 (11.7–1035.3)	246 (18.6–1493)	0.003
	**NAFLD Cirrhosis with HCC (*n* = 21)**	**NAFLD Cirrhosis (*n* = 30)**	***p*-Value**
CXCL5	231.1 (72.4–856.3)	107 (18.4–702.7)	0.008
CXCL9	381.7 (88.8–2325.4)	172 (41.8–3782.2)	0.002
CXCL10	263.5 (131.4–999.5)	125.8 (22.8–736.8)	<0.001
	**ALD Cirrhosis with HCC (*n* = 33)**	**ALD Cirrhosis (*n* = 44)**	***p*-Value**
CCL2	230.2 (19.5–507.1)	145.7 (50.2–778.7)	0.001
CCL25	1,5 (0–32.2)	3.8 (0–166.3)	<0.001
CXCL1	78.9 (15.5–364)	50.9 (4.7–336.2)	0.009
CXCL5	190.5 (44.7–1155.6)	83.8 (14.6–873.4)	0.001
CXCL9	292.9 (121.3–1396.3)	124.8 (29.2–609.4)	<0.001
CXCL10	294.9 (143.7–673.3)	121.3 (24.8–570.4)	<0.001
CXCL11	23 (11.8–50.1)	18.4 (3.9–52.6)	0.023
	**Cholestatic/Autoimmune** **Cirrhosis with HCC (*n* = 4)**	**Cholestatic/Autoimmune** **Cirrhosis (*n* = 14)**	***p*-Value**
CCL3	18.9 (8–28.2)	97.4 (41.7–613.7)	0.003
CXCL5	382.7 (159.9–524.1)	86 (18.9–220.9)	0.003
CXCL10	318.5 (112.9–518.2)	72.8 (26.4–356.8)	0.046
	**HCC Due to Other Etiologies** **(*n* = 8)**	**Cirrhosis Due to Other Etiologies (*n* = 13)**	***p*-Value**
CCL17	390.6 (106.1–595.6)	148.8 (29.8–740.5)	0.002
CXCL1	268.5 (79.6–411.7)	72.9 (37.1–360.9)	0.016
CXCL5	780.3 (565.4–2206-3)	131.8 (14–512.6)	<0.001
CXCL9	391.4 (167.3–2358.8)	88.1 (34.7–887.6)	0.003
CXCL10	248.9 (169.6–456.4)	47.6 (25.8–381)	0.009

Median values are presented with interquartile range in brackets in ng/mL. Abbreviations: ALD, alcoholic liver disease; CCL, chemokine C-C motif ligand; CXCL, C-X-C motif chemokine ligand; HCC, hepatocellular carcinoma; NAFLD, non-alcoholic liver disease.

## Data Availability

The data that support the findings of this study are available from the corresponding author upon reasonable request.

## Abbreviations

AIH	autoimmune hepatitis
ALBI	albumin–bilirubin
ALT	alanine aminotransferase
ALD	alcohol-related liver disease
BCLC	Barcelona Clinic Liver Cancer
CCL	C-C motif chemokine ligand
CXCL	C-X-C motif chemokine ligand
HBC	hepatitis B virus
HCV	hepatitis C virus
HCC	hepatocellular carcinoma
INR	international normalized ratio
IL	interleukin
MELD	model for end-stage liver disease
MVI	macrovascular invasion
NAFLD	non-alcoholic fatty liver disease
PBC	primary biliary cholangitis
PSC	primary sclerosing cholangitis
TME	tumor microenvironment
